# Supplementary material for: Transcriptomic analysis of immune cells in a multi-ethnic cohort of systemic lupus erythematosus patients identifies ethnicity- and disease-specific expression signatures
Source: Commun Biol. 2021 Apr 21;4:488. doi: 10.1038/s42003-021-02000-9 (PMC8060402; doi:10.1038/s42003-021-02000-9)
Supplement: Supplementary file 2 — Supplementary Information [file 42003_2021_2000_MOESM2_ESM.pdf]

Transcriptomic analysis of immune cells in a multi-ethnic cohort of systemic lupus erythematosus patients identifies ethnicity- and disease-specific expression signatures

Gaia Andreoletti[1], Cristina M. Lanata [2], Laura Trupin [2], Ishan Paranjpe[1,3], Tia S. Jain[1], Joanne Nititham[2], Kimberly E. Taylor[2], Alexis J Combes[3], Lenka Maliskova[2], Chun Jimmie Ye[2-4], Patricia Katz[2], Maria Dall'Era[2], Jinoos Yazdany[5], Lindsey A. Criswell[2] & Marina Sirota*[1]

[1]Bakar Computational Health Sciences Institute, University of California, San Francisco, CA, USA. [2]Russell/Engleman Rheumatology Research Center, Department of Medicine, University of California San Francisco, San Francisco, CA, USA.[3] Department of Pathology, University of California, San Francisco, San Francisco, CA 94143, USA; UCSF Immunoprofiler Initiative, University of California, San Francisco, San Francisco, CA 94143, USA.[4]Department of Epidemiology and Biostatistics, University of California, San Francisco, San Francisco, CA, USA, [5]Division of Rheumatology, Department of Medicine at UCSF

*Corresponding author: Marina Sirota marina.sirota@ucsf.edu, ORCID: 0000-0002-7246-6083

Supplementary Figures:

A

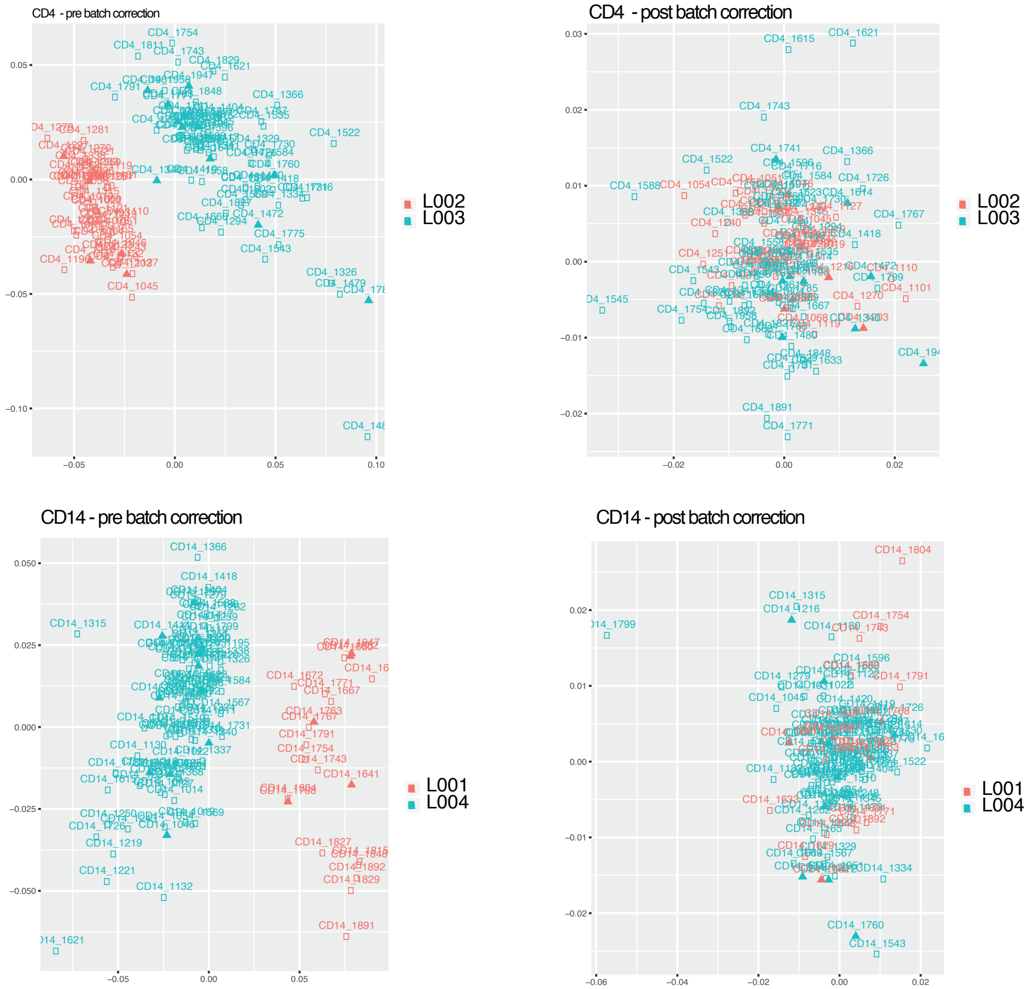

B

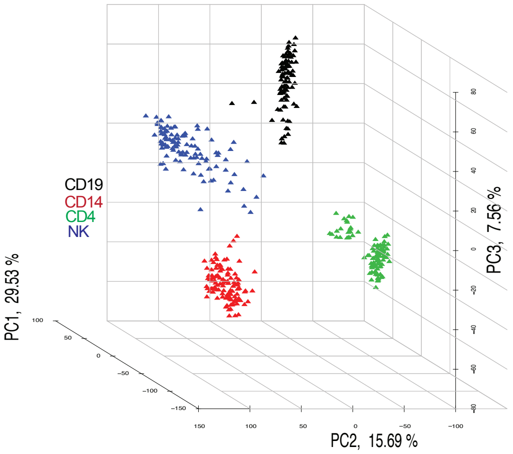

C

Cell	Samples retained	Samples removed	
NK	91	29	
CD19	105	15	
CD14	108	12	
CD4	111	9	
Total samples removed		65	~10% of 480

Supplementary Figure 1: PCA visualization and quality control. A) Data before and after batch correction using limma. Batch effect was observed only in $CD4^+$ T cells and $CD14^+$ monocytes. K-means clustering after batch correction. B) Principal component analysis (PCA) on the batch corrected data. C) Table detailing the number of samples removed per immune-cell type after QC.

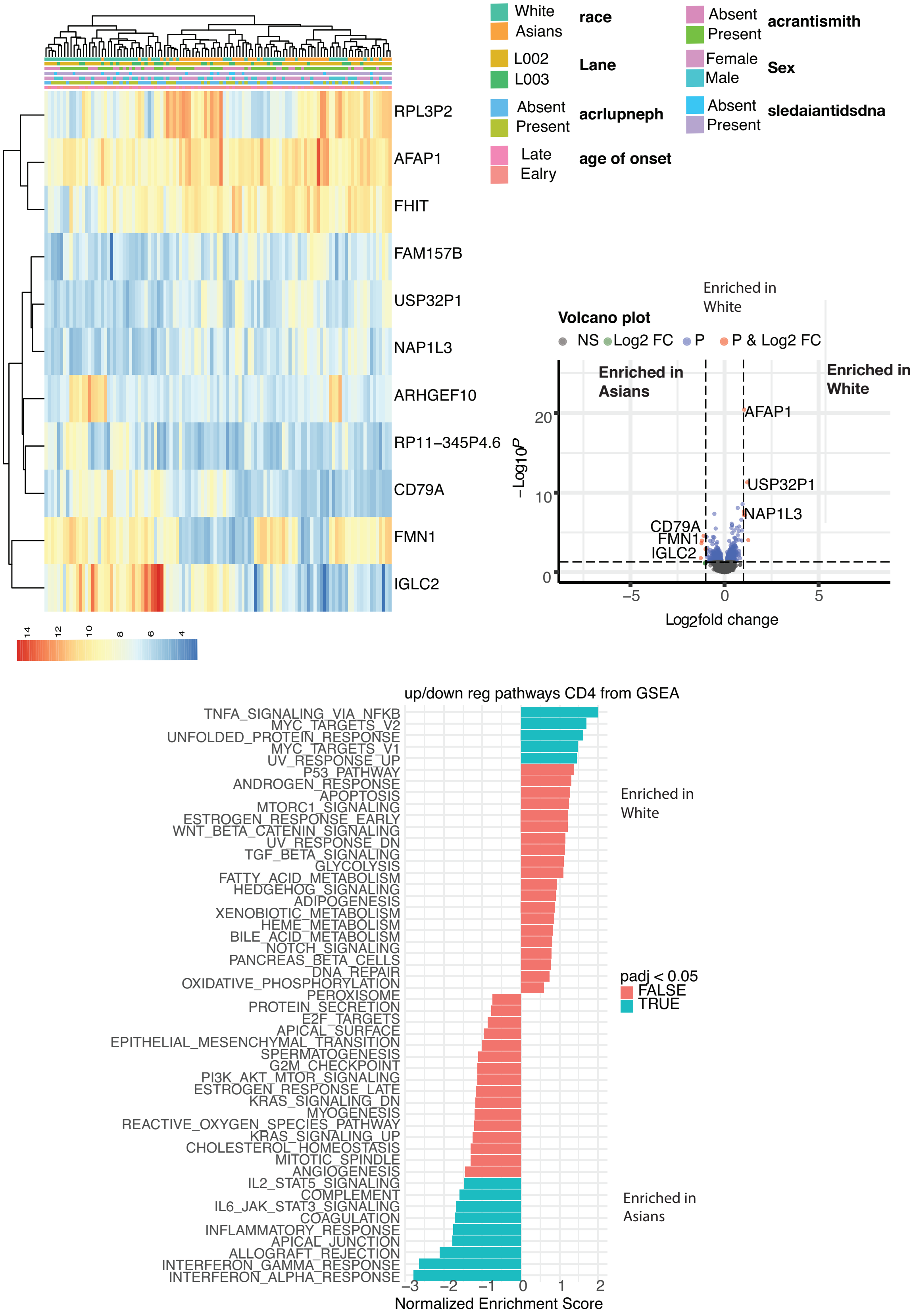

RPL3P2
AFAP1
FHIT
FAM157B
USP32P1
NAP1L3
ARHGEF10
RP11−345P4.6
CD79A
FMN1
IGLC2
14
12
10
8
6
4
White
Asians
race
L002
L003
Lane
Absent
Present
acrlupneph
Late
Ealry
age of onset
Absent
Present
acrantismith
Female
Male
Sex
Absent
Present
sledaiantidsdna
Enriched in White
Volcano plot
NS
Log2 FC
P
P & Log2 FC
Enriched in Asians
Enriched in White
AFAP1
USP32P1
NAP1L3
CD79A
FMN1
IGLC2
20
10
0
−5
0
5
− Log10P
Log2fold change
up/down reg pathways CD4 from GSEA
TNFA_SIGNALING_VIA_NFKB
MYC_TARGETS_V2
UNFOLDED_PROTEIN_RESPONSE
MYC_TARGETS_V1
UV_RESPONSE_UP
P53_PATHWAY
ANDROGEN_RESPONSE
APOPTOSIS
MTORC1_SIGNALING
ESTROGEN_RESPONSE_EARLY
WNT_BETA_CATENIN_SIGNALING
UV_RESPONSE_DN
TGF_BETA_SIGNALING
GLYCOLYSIS
FATTY_ACID_METABOLISM
HEDGEHOG_SIGNALING
ADIPOGENESIS
XENOBIOTIC_METABOLISM
HEME_METABOLISM
BILE_ACID_METABOLISM
NOTCH_SIGNALING
PANCREAS_BETA_CELLS
DNA_REPAIR
OXIDATIVE_PHOSPHORYLATION
PEROXISOME
PROTEIN_SECRETION
E2F_TARGETS
APICAL_SURFACE
EPITHELIAL_MESENCHYMAL_TRANSITION
SPERMATOGENESIS
G2M_CHECKPOINT
PI3K_AKT_MTOR_SIGNALING
ESTROGEN_RESPONSE_LATE
KRAS_SIGNALING_DN
MYOGENESIS
REACTIVE_OXYGEN_SPECIES_PATHWAY
KRAS_SIGNALING_UP
CHOLESTEROL_HOMEOSTASIS
MITOTIC_SPINDLE
ANGIOGENESIS
IL2_STAT5_SIGNALING
COMPLEMENT
IL6_JAK_STAT3_SIGNALING
COAGULATION
INFLAMMATORY_RESPONSE
APICAL_JUNCTION
ALLOGRAFT_REJECTION
INTERFERON_GAMMA_RESPONSE
INTERFERON_ALPHA_RESPONSE
−3
−2
−1
0
1
2
Normalized Enrichment Score
Enriched in White
padj < 0.05
FALSE
TRUE
Enriched in Asians

Supplementary Figure 2: Differential Expression by race for $CD4^+$ T cell. Heatmap, volcano plot and gene set enrichment analyses (GSEA) on the significant genes (FDR < 0.05 and abs(logFC) 1).

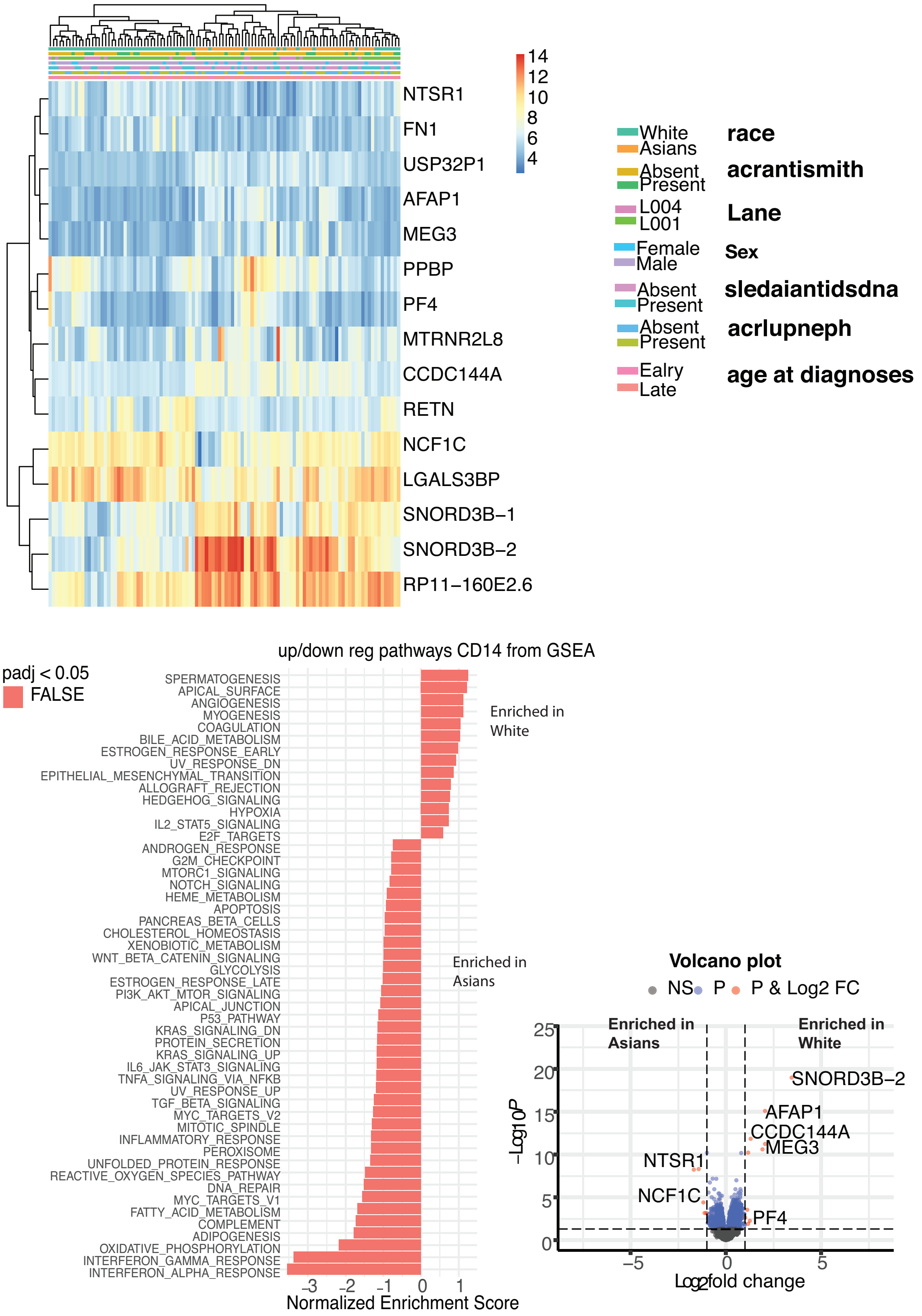

Supplementary Figure 3: Differential Expression by race for CD14⁺ monocytes.

Heatmap, volcano plot and gene set enrichment analyses (GSEA) on the significant genes (FDR < 0.05 and abs(logFC) 1).

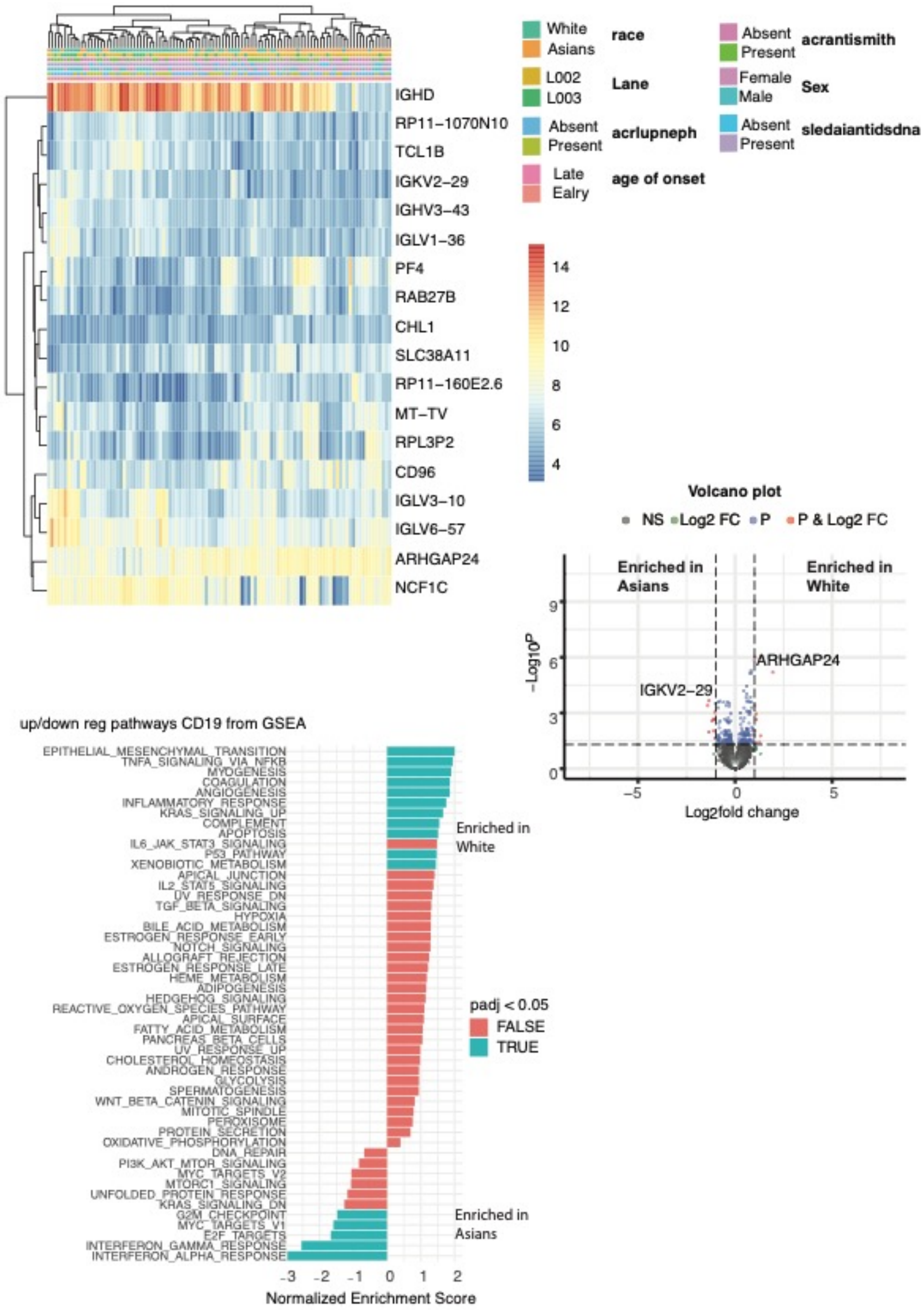

Supplementary Figure 4: Differential Expression by race for CD19 B cell. Heatmap,

volcano plot and gene set enrichment analyses (GSEA) on the significant genes (FDR < 0.05 and abs(logFC) 1).

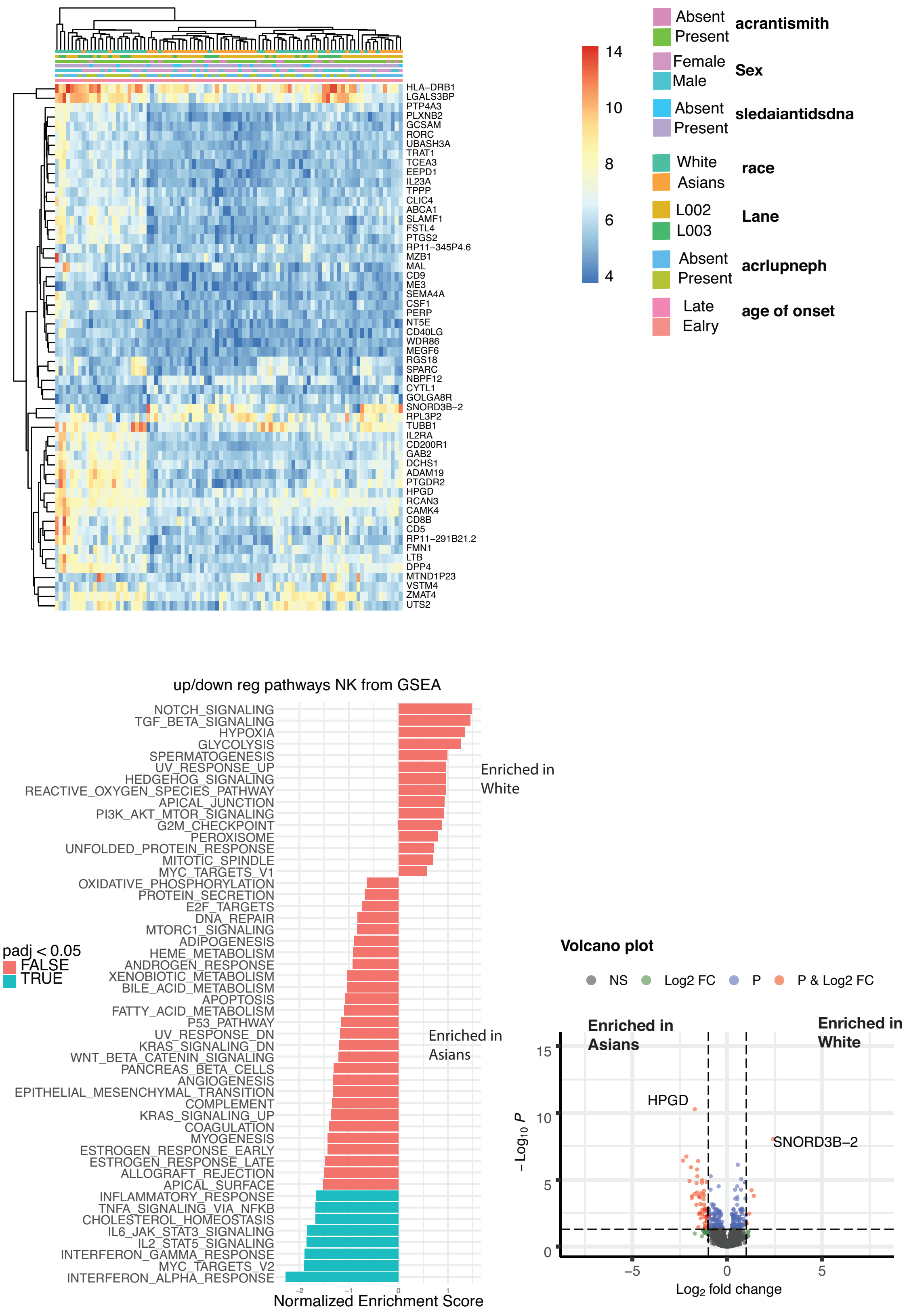

Supplementary Figure 5: Differential Expression by race for NK cells. Heatmap,

volcano plot and gene set enrichment analyses (GSEA) on the significant genes (FDR < 0.05 and abs(logFC) 1).

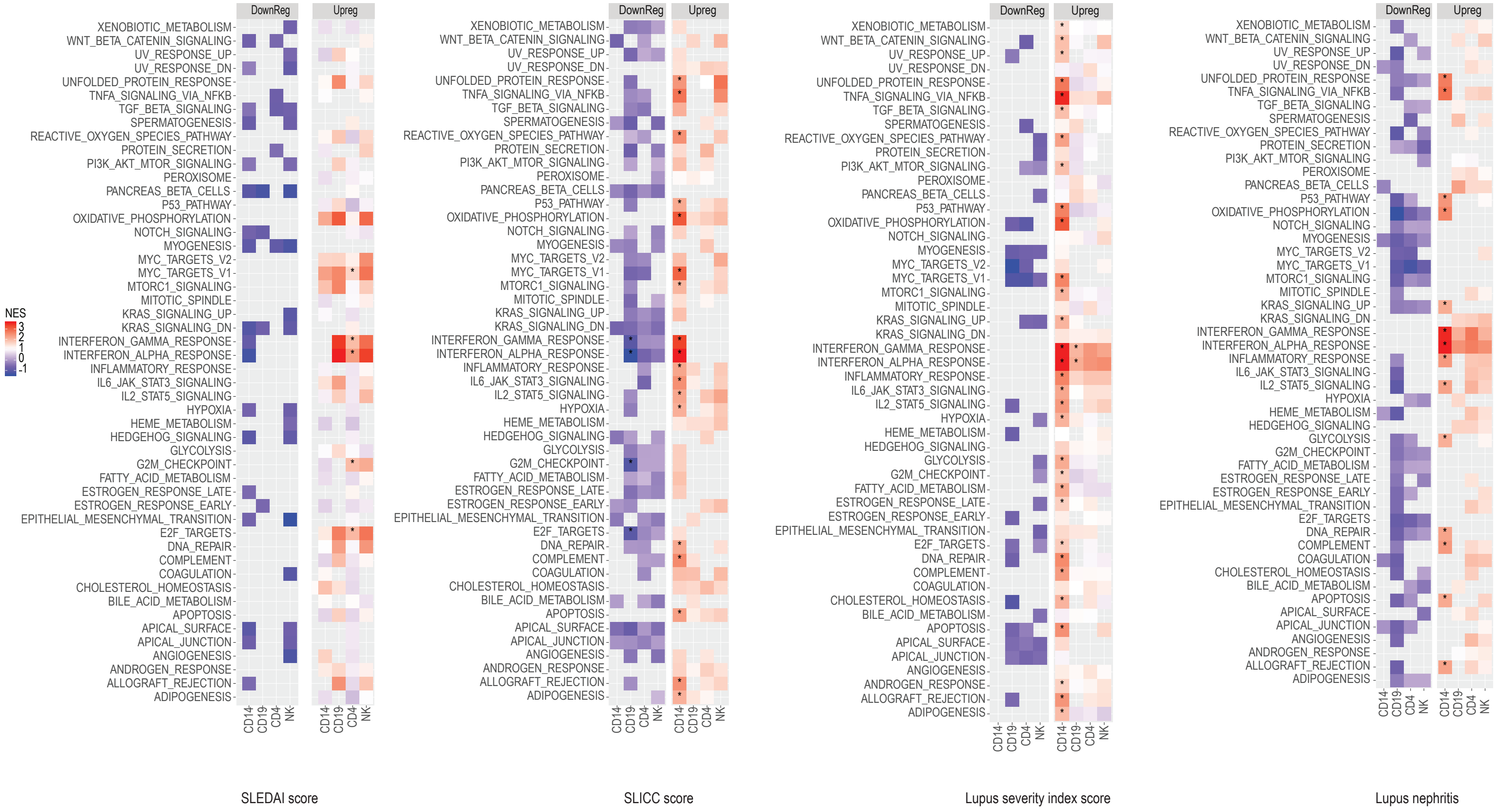

Supplementary Figure 6: Heatmaps displaying pathways analyses across clinical features. Pathway Analysis using GSEA from DE conducted on other clinical criteria Sledai score, slicc score, lupus severity index, and lupus nephritis

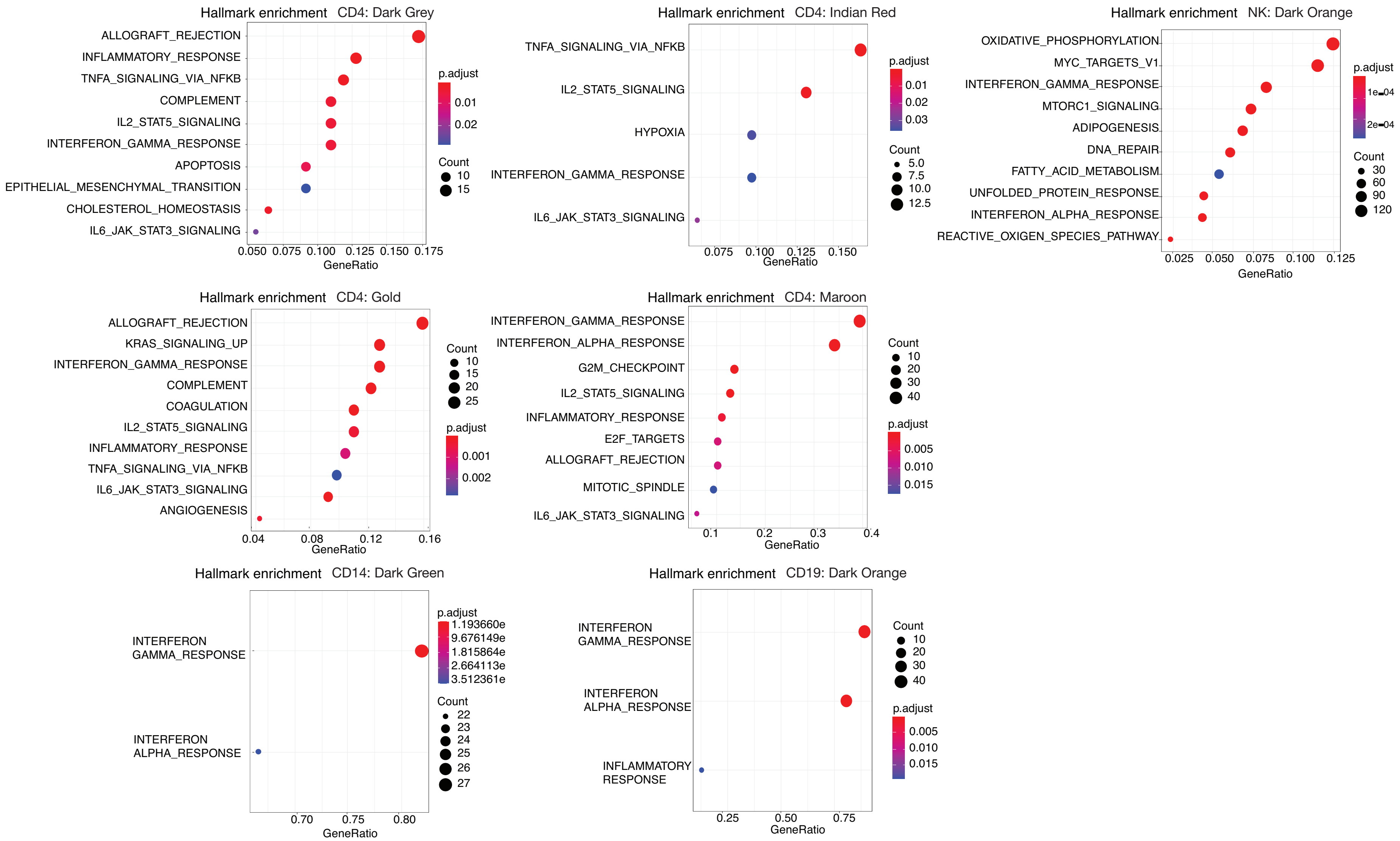

Hallmark enrichment CD4: Dark Grey
ALLOGRAFT_REJECTION
INFLAMMATORY_RESPONSE
TNFA_SIGNALING_VIA_NFKB
COMPLEMENT
IL2_STAT5_SIGNALING
INTERFERON_GAMMA_RESPONSE
APOPTOSIS
EPITHELIAL_MESENCHYMAL_TRANSITION
CHOLESTEROL_HOMEOSTASIS
IL6_JAK_STAT3_SIGNALING
0.050 0.075 0.100 0.125 0.150 0.175
GeneRatio
p.adjust
0.01
0.02
Count
10
15
Hallmark enrichment CD4: Indian Red
TNFA_SIGNALING_VIA_NFKB
IL2_STAT5_SIGNALING
HYPOXIA
INTERFERON_GAMMA_RESPONSE
IL6_JAK_STAT3_SIGNALING
0.075 0.100 0.125 0.150
GeneRatio
p.adjust
0.01
0.02
0.03
Count
5.0
7.5
10.0
12.5
Hallmark enrichment NK: Dark Orange
OXIDATIVE_PHOSPHORYLATION
MYC_TARGETS_V1
INTERFERON_GAMMA_RESPONSE
MTORC1_SIGNALING
ADIPOGENESIS
DNA_REPAIR
FATTY_ACID_METABOLISM
UNFOLDED_PROTEIN_RESPONSE
INTERFERON_ALPHA_RESPONSE
REACTIVE_OXIGEN_SPECIES_PATHWAY
0.025 0.050 0.075 0.100 0.125
GeneRatio
p.adjust
1e-04
2e-04
Count
30
60
90
120
Hallmark enrichment CD4: Gold
ALLOGRAFT_REJECTION
KRAS_SIGNALING_UP
INTERFERON_GAMMA_RESPONSE
COMPLEMENT
COAGULATION
IL2_STAT5_SIGNALING
INFLAMMATORY_RESPONSE
TNFA_SIGNALING_VIA_NFKB
IL6_JAK_STAT3_SIGNALING
ANGIOGENESIS
0.04 0.08 0.12 0.16
GeneRatio
Count
10
15
20
25
p.adjust
0.001
0.002
Hallmark enrichment CD4: Maroon
INTERFERON_GAMMA_RESPONSE
INTERFERON_ALPHA_RESPONSE
G2M_CHECKPOINT
IL2_STAT5_SIGNALING
INFLAMMATORY_RESPONSE
E2F_TARGETS
ALLOGRAFT_REJECTION
MITOTIC_SPINDLE
IL6_JAK_STAT3_SIGNALING
0.1 0.2 0.3 0.4
GeneRatio
Count
10
20
30
40
p.adjust
0.005
0.010
0.015
Hallmark enrichment CD14: Dark Green
INTERFERON GAMMA_RESPONSE
INTERFERON ALPHA_RESPONSE
0.70 0.75 0.80
GeneRatio
p.adjust
1.193660e
9.676149e
1.815864e
2.664113e
3.512361e
Count
22
23
24
25
26
27
Hallmark enrichment CD19: Dark Orange
INTERFERON GAMMA_RESPONSE
INTERFERON ALPHA_RESPONSE
INFLAMMATORY RESPONSE
0.25 0.50 0.75
GeneRatio
Count
10
20
30
40
p.adjust
0.005
0.010
0.015

Supplementary Figure 7: Functional enrichment (Hallmark pathways) of co-expressed modules enriched for interferon pathways

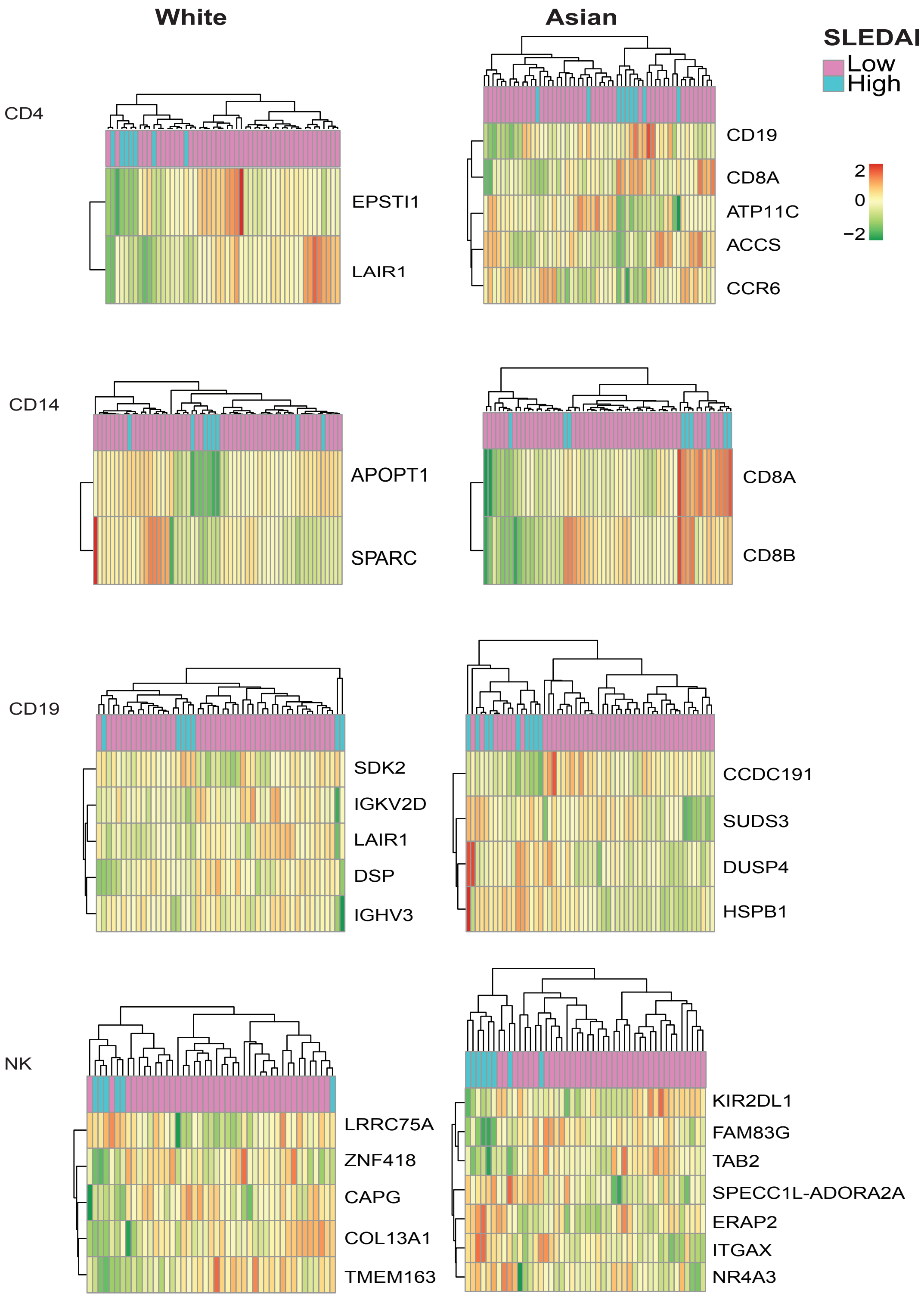

Supplementary Figure 8: Machine learning heatmaps. Clustergrams by cell type for each ethnic group by using the expression values of the top predictors.

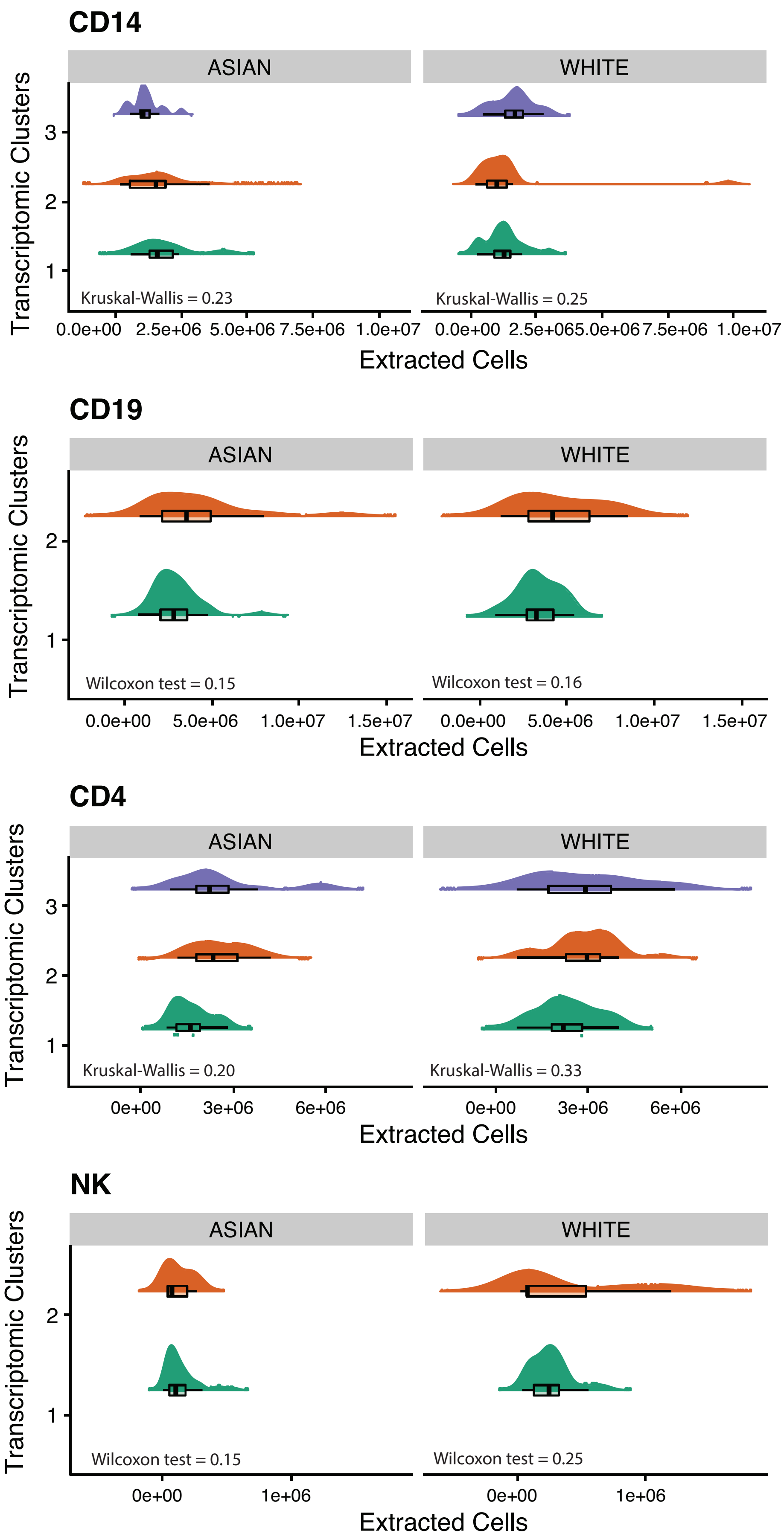
CD14
ASIAN
WHITE
Transcriptomic Clusters
3
2
1
Kruskal-Wallis = 0.23
Kruskal-Wallis = 0.25
0.0e+00 2.5e+06 5.0e+06 7.5e+06 1.0e+07
0.0e+00 2.5e+065.0e+067.5e+06 1.0e+07
Extracted Cells
CD19
ASIAN
WHITE
Transcriptomic Clusters
2
1
Wilcoxon test = 0.15
Wilcoxon test = 0.16
0.0e+00 5.0e+06 1.0e+07 1.5e+07
0.0e+00 5.0e+06 1.0e+07 1.5e+07
Extracted Cells
CD4
ASIAN
WHITE
Transcriptomic Clusters
3
2
1
Kruskal-Wallis = 0.20
Kruskal-Wallis = 0.33
0e+00 3e+06 6e+06
0e+00 3e+06 6e+06
Extracted Cells
NK
ASIAN
WHITE
Transcriptomic Clusters
2
1
Wilcoxon test = 0.15
Wilcoxon test = 0.25
0e+00 1e+06
0e+00 1e+06
Extracted Cells

Supplementary Figure 9: Cell counts distribution visualization. Cell count distribution across the identified transcriptomic clusters for Asian and White Cohorts. None of the distributions are statistically significantly different between groups of interest.

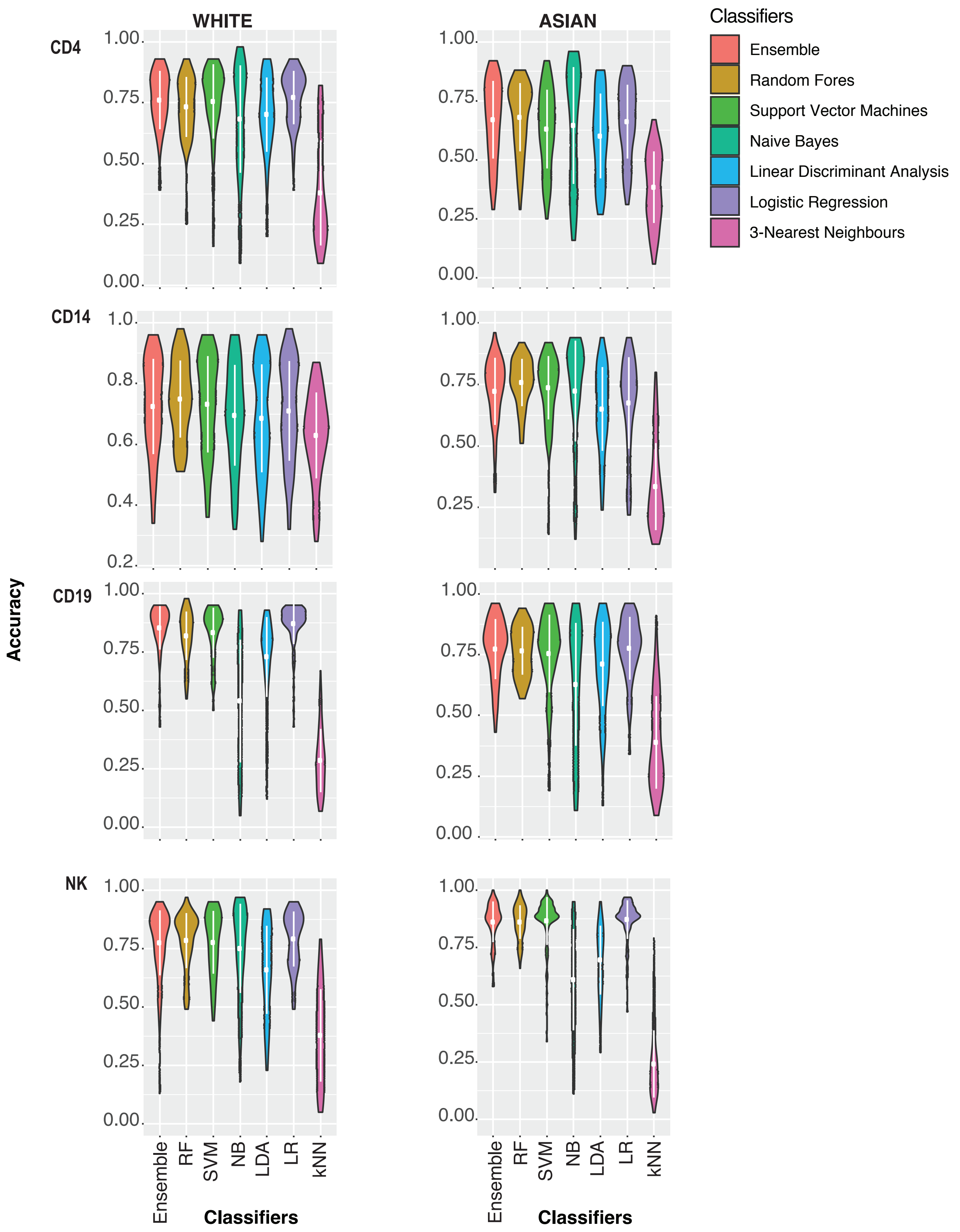
WHITE
ASIAN
CD4
CD14
CD19
NK
Accuracy
Classifiers
Ensemble
Random Fores
Support Vector Machines
Naive Bayes
Linear Discriminant Analysis
Logistic Regression
3-Nearest Neighbours
Ensemble
RF
SVM
NB
LDA
LR
kNN
1.00
0.75
0.50
0.25
0.00
1.0
0.8
0.6
0.4
0.2

Supplementary Figure 10: ML accuracies Comparison. The violin plot highlights the classification accuracy of each classifier, computed at each iteration for each cell line within White and Asian cohorts. Random Forest is the top performer across all and therefore is presented in the main text.

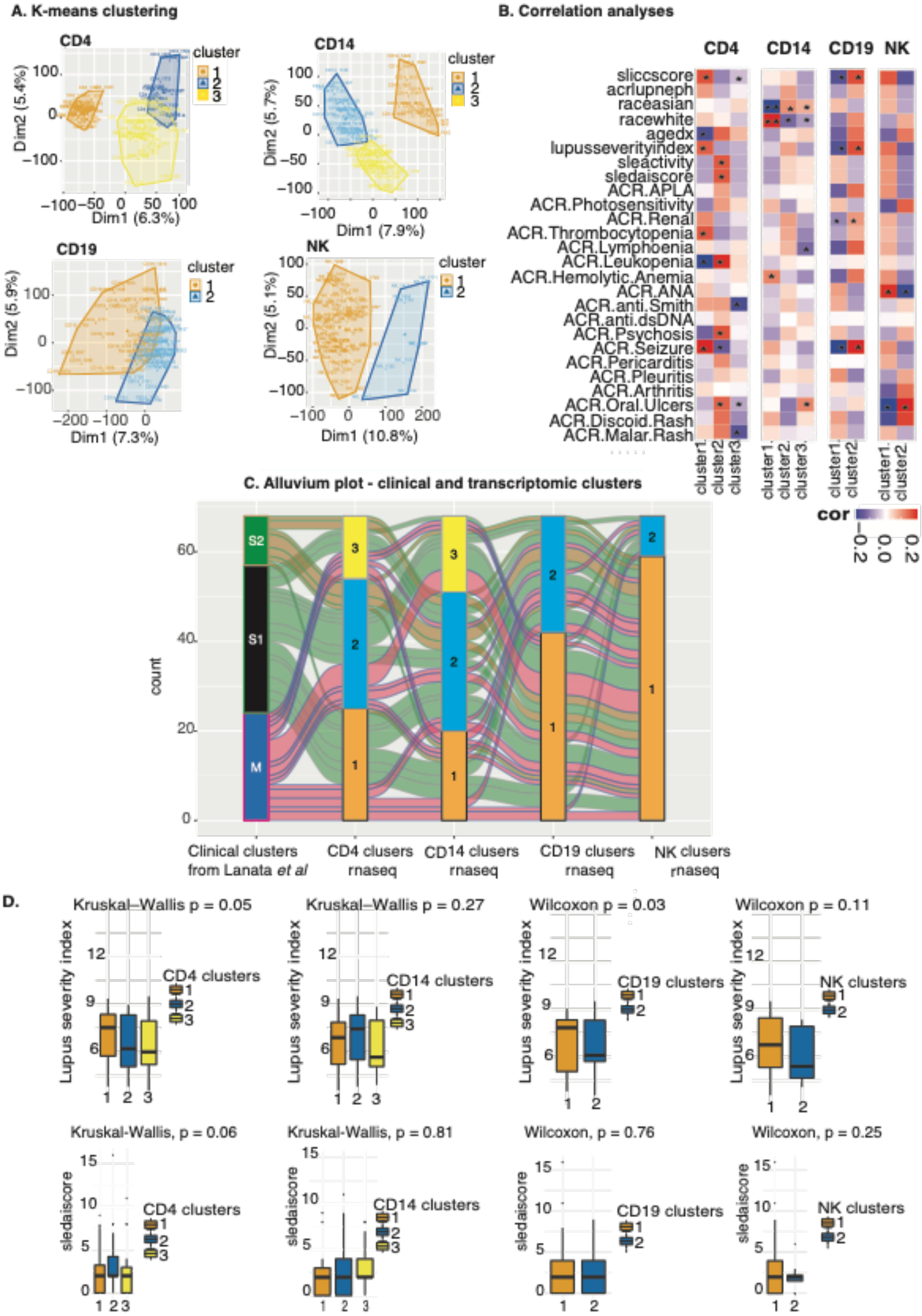
A. K-means clustering
B. Correlation analyses
CD4
CD14
CD19
NK
cluster
Dim1 (6.3%)
Dim2 (5.4%)
Dim1 (7.9%)
Dim2 (5.7%)
Dim1 (7.3%)
Dim2 (5.9%)
Dim1 (10.8%)
Dim2 (5.1%)
sliccscore
acrlupneph
raceasian
racewhite
agedx
lupusseverityindex
sleactivity
sledaiscore
ACR.APLA
ACR.Photosensitivity
ACR.Renal
ACR.Thrombocytopenia
ACR.Lymphoenia
ACR.Leukopenia
ACR.Hemolytic.Anemia
ACR.ANA
ACR.anti.Smith
ACR.anti.dsDNA
ACR.Psychosis
ACR.Seizure
ACR.Pericarditis
ACR.Pleuritis
ACR.Arthritis
ACR.Oral.Ulcers
ACR.Discoid.Rash
ACR.Malar.Rash
cluster1
cluster2
cluster3
cor
C. Alluvium plot - clinical and transcriptomic clusters
count
S2
S1
M
Clinical clusters from Lanata et al
CD4 clusers rnaseq
CD14 clusers rnaseq
CD19 clusers rnaseq
NK clusers rnaseq
D.
Kruskal–Wallis p = 0.05
Kruskal–Wallis p = 0.27
Wilcoxon p = 0.03
Wilcoxon p = 0.11
Lupus severity index
CD4 clusters
CD14 clusters
CD19 clusters
NK clusters
Kruskal-Wallis, p = 0.06
Kruskal-Wallis, p = 0.81
Wilcoxon, p = 0.76
Wilcoxon, p = 0.25
sledaiscore

Supplementary Figure 11: Sensitivity analyses without patients with ACR criteria < 4 and a confirmed diagnoses of lupus nephritis (n=21): A) K-means clustering on the $CD14^{+}$ monocytes, $CD4^{+}$ T-cells, B cells and NK cells. B) Association analyses between clinical parameters and K-means s clustering. Red – positive association, blue – negative association. Number of stars indicate the level of significance. C) Alluvium plot visualizing the distribution of the samples according to different clusters. D) Distribution of lupus severity index and SLEDAI score across clusters with p-value computed using non-parametric tests. Findings between the sensitivity analysis and the main analysis are consistent.

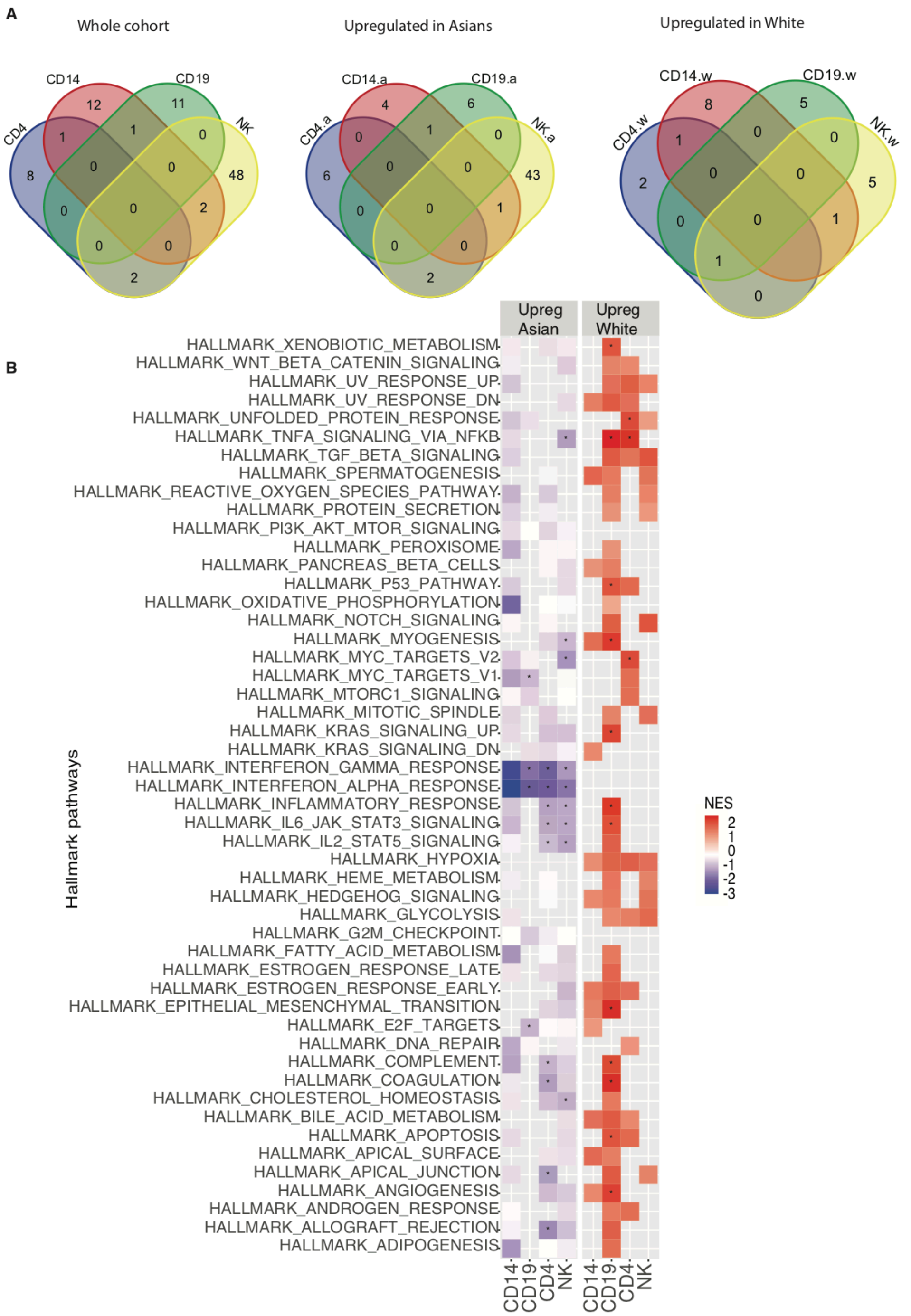

Supplementary Figure 12: Sensitivity analyses without patients with ACR criteria < 4 and a confirmed diagnoses of lupus nephritis: A) Identification of the common DE genes (padj < 0.05 & abs log2FC > 1) for White vs Asian cohorts across the different cell types. B)

Pathway enrichment analyses for the associated genes of White vs Asian cohorts. Findings between the sensitivity analysis and the main analysis are consistent.
