# Supplementary material for: Transcriptomic analysis of immune cells in a multi-ethnic cohort of systemic lupus erythematosus patients identifies ethnicity- and disease-specific expression signatures
Source: Commun Biol. 2021 Apr 21;4:488. doi: 10.1038/s42003-021-02000-9 (PMC8060402; doi:10.1038/s42003-021-02000-9)
Supplement: Supplementary file 3 — Description of Additional Supplementary Files [file 42003_2021_2000_MOESM3_ESM.pdf]

Description of Additional Supplementary Files

File name: Supplementary Data

Description:

Supplementary Data 1: CLUES Cohort Demographics.
*P value done with chi-squared or ANOVA were appropriate. All are ACR clinical feature are a yes/no variables, except flare, which has 3 levels (mild 1, moderate 2, severe 3). N positive= number of individuals having the clinical feature.

Supplementary Data 2: Differential expression analyses genes.
Shared genes across cell types in the differential expression analysis results for White vs Asians in the (a) whole cohort, (b) Asian cohort, and (c) White cohort. Only shared genes with an adjusted p value less than 0.05 and logFC 1 shown.

Supplementary Data 3: Stability index.
Jaccard stability index done on *100 bootstrap iterations

Supplementary Data 4: Clinical and demographic variables across CD4 clusters.
Flare severity measurement: mild 1, moderate 2, severe 3. All are ACR clinical feature are a yes/no variables, except flare, which has 3 levels (mild 1, moderate 2, severe 3). N positive= number of individuals having the clinical feature.

Supplementary Data 5: clinical and demographic variables across CD14 clusters.
Flare severity measurement: mild 1, moderate 2, severe 3 . All are ACR clinical feature are a yes/no variables, except flare, which has 3 levels (mild 1, moderate 2, severe 3). N positive= number of individuals having the clinical feature.

Supplementary Data 6: clinical and demographic variables across CD19 clusters.

Flare severity measurement: mild 1, moderate 2, severe 3. All are ACR clinical feature are a yes/no variables, except flare, which has 3 levels (mild 1, moderate 2, severe 3). N positive= number of individuals having the clinical feature.

Supplementary Data 7: clinical and demographic variables across NK clusters

Flare severity measurement: mild 1, moderate 2, severe 3. All are ACR clinical feature are a yes/no variables, except flare, which has 3 levels (mild 1, moderate 2, severe 3). N positive= number of individuals having the clinical feature.

Supplementary Data 8: Differential gene expression results.

Number of statistically significant genes (p value adjusted < 0.05 & abs (log2FC) 1) for differential expression analyses conducted on lupus severity score, SLICC score, SLEDAI score, Race (White vs Asians), Lupus nephritis, and Age at diagnoses (late vs early onset).
