# Supplementary material for: Transcriptomic analysis of immune cells in a multi-ethnic cohort of systemic lupus erythematosus patients identifies ethnicity- and disease-specific expression signatures
Source: Commun Biol. 2021 Apr 21;4:488. doi: 10.1038/s42003-021-02000-9 (PMC8060402; doi:10.1038/s42003-021-02000-9)
Supplement: Supplementary file 5 — Reporting Summary [file 42003_2021_2000_MOESM5_ESM.pdf]

nature research

Corresponding author(s): COMMSBIO-20-3281B

Last updated by author(s): Feb 21, 2021

Reporting Summary

Nature Research wishes to improve the reproducibility of the work that we publish. This form provides structure for consistency and transparency in reporting. For further information on Nature Research policies, see our Editorial Policies and the Editorial Policy Checklist.

Statistics

For all statistical analyses, confirm that the following items are present in the figure legend, table legend, main text, or Methods section.

n/a	Confirmed	
☐	☒	The exact sample size (*n*) for each experimental group/condition, given as a discrete number and unit of measurement
☐	☒	A statement on whether measurements were taken from distinct samples or whether the same sample was measured repeatedly
☐	☒	The statistical test(s) used AND whether they are one- or two-sided<br>*Only common tests should be described solely by name; describe more complex techniques in the Methods section.*
☐	☒	A description of all covariates tested
☐	☒	A description of any assumptions or corrections, such as tests of normality and adjustment for multiple comparisons
☒	☐	A full description of the statistical parameters including central tendency (e.g. means) or other basic estimates (e.g. regression coefficient) AND variation (e.g. standard deviation) or associated estimates of uncertainty (e.g. confidence intervals)
☒	☐	For null hypothesis testing, the test statistic (e.g. *F*, *t*, *r*) with confidence intervals, effect sizes, degrees of freedom and *P* value noted<br>*Give P values as exact values whenever suitable.*
☒	☐	For Bayesian analysis, information on the choice of priors and Markov chain Monte Carlo settings
☐	☒	For hierarchical and complex designs, identification of the appropriate level for tests and full reporting of outcomes
☐	☒	Estimates of effect sizes (e.g. Cohen's *d*, Pearson's *r*), indicating how they were calculated

Our web collection on statistics for biologists contains articles on many of the points above.

Software and code

Policy information about availability of computer code

Data collection	Participants were recruited from the California Lupus Epidemiology Study (CLUES). CLUES was approved by the Institutional Review Board of the University of California, San Francisco. All participants signed a written informed consent to participate in the study.
Data analysis	Peripheral blood mononuclear cells were isolated from patient donors. Cells were isolated from peripheral blood utilizing magnetic beads (CD14+monocytes, B cells, CD4+T cells and NK cells) using EasySep protocol from STEM cell technologies on 120 patients. RNA and DNA were extracted using Qiagen column and quality was assessed on Agilent bioanalyzer. 1ng - 5ng of RNA was used with SmartSeqv2 protocol to get cDNA, followed by Illumina Nextera XT DNA library prep with input of 0.8ng cDNA and 15 cycles of PCR 20 amplification. Both cDNA and DNA libraries qualities were controlled on Agilent bioanalyzer. Libraries were sequenced on HiSeq4000 PE150. Salmon v0.8.2 was used for our alignment-free pipeline. Adaptor-trimmed reads were used as input. We quantified gene expression using raw counts and kept the genes that showed an average FPKM value across the samples >0.5. FastQC (v0.11.8) was run to assess the quality of the sequence reads. To further assess for low quality samples we checked the expression of 10 housekeeping genes (e.g. SNRPD3, EMC7, and VPS29) called the Eisenberg housekeeping genes16. Finally, we conducted K-means clustering and removed all outlier samples that were > 1 standard deviation from the center of the cluster. The Davies-Bouldin’s index was also computed to confirm cluster stability. Batch correction was performed with limma (v 3.40.6).

For manuscripts utilizing custom algorithms or software that are central to the research but not yet described in published literature, software must be made available to editors and reviewers. We strongly encourage code deposition in a community repository (e.g. GitHub). See the Nature Research guidelines for submitting code & software for further information.

Data

Policy information about availability of data

All manuscripts must include a data availability statement. This statement should provide the following information, where applicable:

- Accession codes, unique identifiers, or web links for publicly available datasets
- A list of figures that have associated raw data
- A description of any restrictions on data availability

The data of this study are openly available in GEO: GSE164457. R custom code used to generate the figures and analysis can be found on figshare https://rb.gy/mhyafy

Field-specific reporting

Please select the one below that is the best fit for your research. If you are not sure, read the appropriate sections before making your selection.

- [x] Life sciences
- [ ] Behavioural & social sciences
- [ ] Ecological, evolutionary & environmental sciences

For a reference copy of the document with all sections, see nature.com/documents/nr-reporting-summary-flat.pdf

Life sciences study design

All studies must disclose on these points even when the disclosure is negative.

Sample size	120 SLE patients - 4 cells lines for a total of 480 samples.
Data exclusions	samples were removed if 1) the expression of 10 housekeeping genes (e.g. SNRPD3, EMC7, and VPS29) called the Eisenberg housekeeping genes was low. 2) we conducted K-means clustering and removed all outlier samples that were > 1 standard deviation from the center of the cluster. The Davies-Bouldin's index was also computed to confirm cluster stability. Batch correction was performed with limma (v 3.40.6
Replication	N/A
Randomization	samples were divided based on self reported ethnicity (asians vs white) and based on disease activity (based on SLEDAI score)
Blinding	Blinding was outside the scope of this study

Reporting for specific materials, systems and methods

We require information from authors about some types of materials, experimental systems and methods used in many studies. Here, indicate whether each material, system or method listed is relevant to your study. If you are not sure if a list item applies to your research, read the appropriate section before selecting a response.

Materials & experimental systems

n/a	Involved in the study	
[x]	[ ]	Antibodies
[x]	[ ]	Eukaryotic cell lines
[x]	[ ]	Palaeontology and archaeology
[x]	[ ]	Animals and other organisms
[ ]	[x]	Human research participants
[x]	[ ]	Clinical data
[x]	[ ]	Dual use research of concern

Methods

n/a	Involved in the study	
[x]	[ ]	ChIP-seq
[x]	[ ]	Flow cytometry
[x]	[ ]	MRI-based neuroimaging

Human research participants

Policy information about studies involving human research participants

Population characteristics	Systemic lupus erythematosus (SLE) is a heterogeneous autoimmune disease in which outcomes vary among different racial groups. The aim of this study is to leverage large-scale transcriptomic data from diverse populations to better sub-classify SLE patients into more clinically actionable groups. We leverage cell sorted RNA-seq data (CD14+ monocytes, B cells, CD4+T cells, and NK cells) from 120 SLE patients (63 Asian and 57 White individuals) and apply a four tier analytical approach to identify SLE subgroups within this multiethnic cohort: unsupervised clustering, differential expression analyses, gene co-expression analyses, and machine learning.
Recruitment	Participants were recruited from the California Lupus Epidemiology Study (CLUES). CLUES was approved by the Institutional Review Board of the University of California, San Francisco. All participants signed a written informed consent to participate in the study. Study procedures involved an in-person research clinic visit, which included collection and review of medical records prior to the visit; a history and physical examination conducted by a physician specializing in lupus; collection of biospecimens, including peripheral blood for clinical and research purposes; and completion of a structured interview administered by an experienced research assistant. All SLE diagnoses were confirmed by study physicians based upon one of the following definitions: (a) meeting≥4 of the 11 American College of Rheumatology (ACR) revised criteria for the classification of SLE as defined in 1982 and updated in 1997, (b) meeting 3 of the 11 ACR criteria plus a documented rheumatologist's diagnosis of SLE, or (c) a confirmed diagnosis of lupus nephritis, defined as fulfilling the ACR renal classification criterion (>0.5 grams of proteinuria per day or 3+protein on urine dipstick analysis) or having evidence of lupus nephritis on kidney biopsy. A total of 120 patients were profiled with bulk RNA-seq from the CLUES cohort.
Ethics oversight	CLUES was approved by the Institutional Review Board of the University of California, San Francisco.

Note that full information on the approval of the study protocol must also be provided in the manuscript.
